# Guidelines for the management of *Helicobacter pylori* infection in Japan: 2024 revised edition

**DOI:** 10.1007/s00535-026-02379-4

**Published:** 2026-03-28

**Authors:** Hajime Isomoto, Tadashi Shimoyama, Masanori Ito, Takako Osaki, Osamu Toyoshima, Juntaro Matsuzaki, Hiroyoshi Ota, Daisuke Chinda, Mitsushige Sugimoto, Soichiro Sue, Koji Otani, Toshihiro Nishishizawa, Osamu Handa, Takahisa Furuta, Hideki Mori, Masumi Okuda, Yuichiro Ikebuchi, Kazuhiko Inoue, Kyoko Sakai, Yuji Nadatani, Ryota Niikura, Kazuo Yashima, Tamaki Ikuse, Toshihiko Kakiuchi, Momoko Tsuda, Katsuhiro Mabe, Yasuhiro Mizuno, Kazunari Murakami, Hidekazu Suzuki

**Affiliations:** 1 Department of Gastroenterology and Nephrology, Tottori University School of Medicine, Yonago, Japan; 2 Aomori General Health Examination Center, Aomori, Japan; 3 Department of General Internal Medicine, Hiroshima University Hospital, Hiroshima, Japan; 4 Department of Infectious Diseases, Kyorin University School of Medicine, Mitaka, Japan; 5 Gastroenterology, Toyoshima Endoscopy Clinic, Tokyo, Japan; 6 Keio University Faculty of Pharmacy, Tokyo, Japan; 7 Department of Clinical Laboratory Sciences, Shinshu University School of Medicine, Matsumoto, Japan; 8 Division of Endoscopy, Hirosaki University Hospital, Hirosaki, Japan; 9 Division of Genome-Wide Infectious Microbiology, Research Center for GLOBAL and LOCAL Infectious Disease, Oita University, Yufu, Japan; 10 Department of Gastroenterology, Yokohama City University Graduate School of Medicine, Yokohama, Japan; 11 Department of Gastroenterology, Osaka Metropolitan University Graduate School of Medicine, Osaka, Japan; 12 Department of Gastroenterology and Hepatology, International University of Health and Welfare Narita Hospital, Narita, Japan; 13 Department of Gastroenterology and Hepatology, Kawasaki Medical School, Kurashiki, Japan; 14 Furuta Clinic for Internal Medicine, Iwata, Japan; 15 Division of Gastroenterology and Hepatology, Department of Internal Medicine, Keio University School of Medicine, Tokyo, Japan; 16 Department of Pediatrics, Hyogo Medical University, Nishinomiya, Japan; 17 Motomachi Hospital, Sakaiminato, Japan; 18 Junpukai Health Maintenance Center, Okayama, Japan; 19 Department of Laboratory Medicine, Suita Saiseikai Hospital, Suita, Japan; 20 Department of Health Informatics, School of Public Health, Kyoto University, Kyoto, Japan; 21 Department of Advanced Preventive Medicine, Osaka Metropolitan University Graduate School of Medicine, Osaka, Japan; 22 Preventive Medicine Center, Tokyo Medical University, Tokyo, Japan; 23 Department of Pediatrics, Juntendo University Faculty of Medicine, Tokyo, Japan; 24 Department of Pediatrics, Faculty of Medicine, Saga University, Saga, Japan; 25 Department of Gastroenterology, Sapporo Cancer Screening Center, Public Interest Foundation Hokkaido Cancer Society, Sapporo, Japan; 26 Mabe Goryokaku Gastrointestinal Endoscopy Clinic, Hakodate, Japan; 27 Ma-Ru Clinic Yokosuka, Yokosuka, Japan; 28 Department of Gastroenterology, Oita University School of Medicine, Yufu, Japan; 29 Division of Gastroenterology and Hepatology, Department of Internal Medicine, Tokai University School of Medicine, Isehara, Kanagawa 259-1193 Japan

**Keywords:** *Helicobacter pylori*, Diagnosis, Eradication, Gastric cancer prevention, Clinical questions

## Abstract

**Background:**

The Japanese Society for Helicobacter Research (JSHR) published the fifth edition of the guidelines for the management of *Helicobacter pylori* (*H. pylori*) infection in 2024 in Japanese language.

**Methods:**

This edition of JSHR guidelines was first developed in accordance with the Minds Clinical Practice Guideline and Grading of Recommendations Assessment, Development, and Evaluation system. This was published after inviting public comments and external evaluations. Consensus on clinical questions (CQs) was achieved using the modified Delphi method.

**Results:**

There was no change in the basic policy that all *H. pylori* infectious diseases are indications for eradication. Nucleic acid amplification was added to the diagnostic methods. Effects of proton-pump inhibitors (PPIs) on diagnostic tests were updated. The superiority of potassium-competitive acid blockers to PPIs in 1-week triple therapy with amoxicillin and clarithromycin has been shown. A flowchart for eradication therapy was provided for use in real-world settings. For gastric cancer prevention, eradication in earlier age is recommended. The most appropriate test and treatment strategies for adolescents are also described. Some CQs concerning gastric cancer prevention were identified, although evidence level was insufficient to make recommendations based on clinical importance.

**Conclusion:**

The revised guidelines facilitate the appropriate management of *H. pylori* infection and gastric cancer prevention.

## Introduction

The fifth revision (2024) of the *Guidelines for the Diagnosis and Treatment of Helicobacter pylori Infection,* published by The Japanese Society for Helicobacter Research (JSHR), was developed fully in accordance with the *Minds (Medical Information Network Distribution Service) Clinical Practice Guideline Development Manual 2020, ver. 3.0* [1]. Evidence was appraised using the Grading of Recommendations Assessment, Development, and Evaluation (GRADE) framework, which categorizes evidence quality into four levels (A‒D) and recommendations as either “Strong” or “Weak” [2]. The predetermined agreement level was defined as 70% in the guidelines. Consensus on clinical questions (CQs) was achieved through a modified Delphi process and a nominal group technique, requiring the participation of at least two-thirds of committee members. Each CQ underwent structured voting using five response options, and the recommendations were finalized according to predefined thresholds. Limited yet clinically meaningful evidence, such as non–*Helicobacter pylori Helicobacter* species, was retained rather than reclassified as future research questions (FRQs). The guidelines provided recommendations based on scientific evidence, with the validity of each medical practice related to *Helicobacter pylori* (*H. pylori*) infection management, and several CQs included recommendations for therapies or tests that are not covered by the national health insurance system in Japan. The primary objective of the guidelines is not merely to reflect current routine clinical practice or insurance coverage but rather to present optimal diagnostic and therapeutic strategies based on scientific evidence. When considering such non-covered medical practices, approval from the institution’s ethics committee is required in each practical setting. It is also necessary to ensure that decisions are made by specialists for the implementation of the most appropriate treatment while considering the patients’ values and preferences as needed.

Updates in the current revision include the reassessment of diagnostic accuracy during proton-pump inhibitor (PPI) use, incorporation of potassium-competitive acid blockers (P-CABs) in first-line eradication therapy, and recommendation of drug susceptibility testing prior to eradication therapy. The most updated and informative flowchart of *H. pylori* therapy is shown. The 2024 guidelines also highlight the importance of early detection and eradication of *H. pylori* infection during junior high school as a preventive strategy against gastric cancer. Furthermore, the guidelines retain best-practice CQs supported by limited evidence, including data from single-arm studies, thereby maintaining a focus on pediatric and adolescent management while anticipating future updates as new data become available.

This article provides an overview of each CQ across four domains—diagnosis, treatment, and prevention of gastric cancer in adults as well as adolescent populations—and includes selected background questions (BQ) within the diagnostic and therapeutic sections.

## Diagnosis

### Selected BQs in diagnosis

**Are gastroscopy images useful for diagnosing current** ***H. pylori*** **infection?**

- Endoscopic images can classify *H. pylori* infection status, making them useful for diagnosing current infections.

**Fig. 1:**
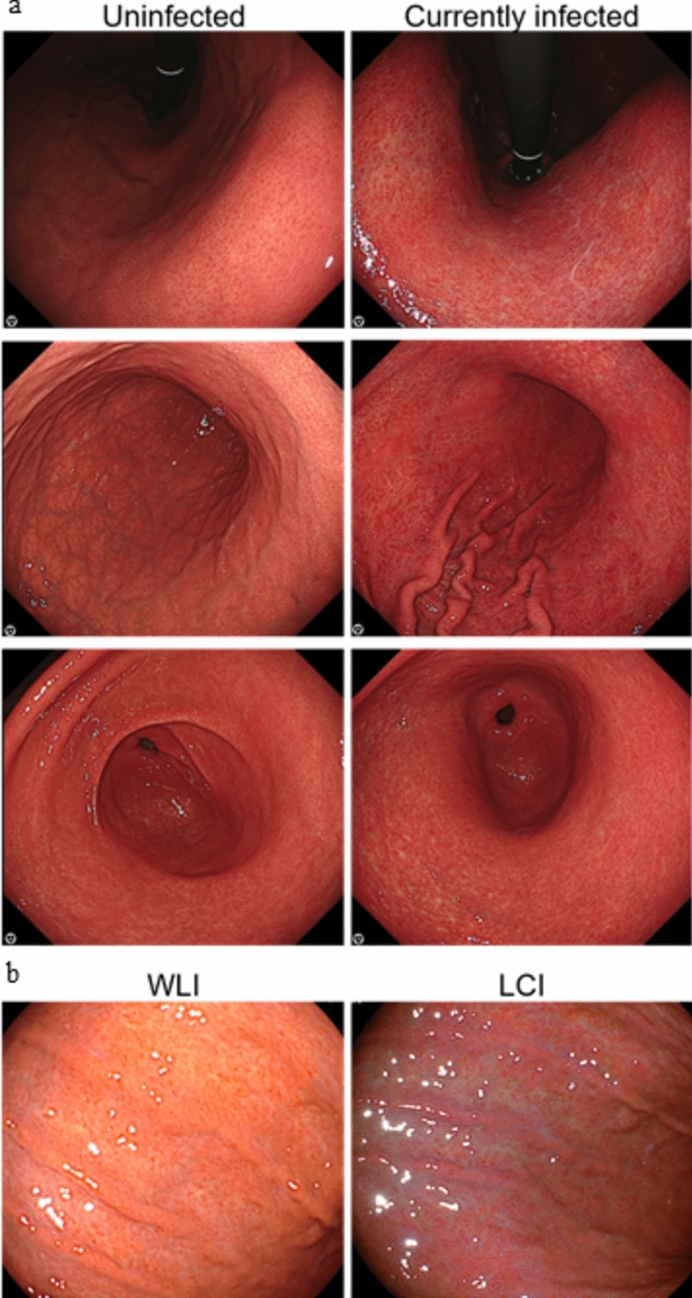
Endoscopic images diagnosing current *H. pylori* infection. **a** Comparison of stomachs with and without current *Helicobacter pylori* (*H. pylori*) infection. Diffuse redness, mucosal swelling, enlarged folds, and sticky mucus were observed in the stomach after *H. pylori* infection. Images were obtained using the EVIS X1 endoscopic system with GIF-XZ1200 (Olympus. Co., Tokyo, Japan). **b** Comparison of white light imaging (WLI) and linked color imaging (LCI). Images were obtained using the ELUXEO 7000 endoscopic system with EG-760Z (Fujifilm Medical Co., Tokyo, Japan)

Comment: In 2014, the endoscopy-based Kyoto Classification of Gastritis was established to assess *H. pylori* infection and gastric cancer risk [3]. For current infection, it identifies diffuse redness (loss of regular arrangement of collecting venules) and mucosal swelling as “frequently observed,” whereas enlarged folds and sticky mucus are “observed” (Fig. 1a). Yoshii et al. quantified the diagnostic odds ratios for these findings and found that diffuse redness (26.8) was the strongest predictor, followed by mucosal swelling (13.3), sticky mucus (10.2), and enlarged folds (8.6) [4]. Improvement in diffuse redness is an indicator of successful eradication. Recent advances in Linked Color Imaging (linked color imaging) have enhanced sensitivity for detecting *H. pylori* infection by effectively highlighting features such as diffuse redness (Fig. 1b) [5].

**What is important for the accurate diagnosis of** ***H. pylori*** **infection, particularly during administration of PPIs?**

- Use of multiple diagnostic tests is recommended to diagnose *H. pylori* infection accurately.
- Urea breath test (UBT), rapid urease test (RUT), and pepsinogen (PG) levels cannot be used during PPI.

Comment: No diagnostic test has 100% sensitivity or 100% specificity. Diagnostic test results should be interpreted based on the endoscopic findings of the gastric mucosa [6]. Additional tests should be performed if the diagnostic test results are inconclusive. Since PPI administration suppresses *H. pylori* urease activity, UBT and RUT are not reliable during PPI therapy [6]. In contrast, the domestic stool antigen test (SAT) based on a monoclonal antibody can be used during PPI administration after local validation [7, 8]. Nucleic acid amplification test (NAAT) using intragastric fluid is also not influenced by PPI administration (Table 1) [9]. Both UBT and SAT are recommended for evaluating eradication therapy results [6].

**Table 1:** PPI administration and diagnostic tests of *H. pylori* infection

	Can continue PPI administration	Stop PPI for 2 weeks prior
Invasive	Histology, culture	Rapid urease test (RUT)
Non-invasive	Serum or urine antibody Stool antigen test (SAT) Nucleic acid amplification test (NAAT)	Urea breath test (UBT)
Supporting test		Serum pepsinogen level

### CQ1-1

**Is UBT recommended for diagnosing** ***H. pylori*** **infection?**

- UBT is recommended for diagnosing *H. pylori* infection before and after eradication.

(Recommendation: strong (100% strong agreement), evidence level B).

Comment: Appropriate cutoff values for UBT in diagnosing *H. pylori* infection before and after eradication are 2.5‒3.5‰ and 3.5‒4.0‰, respectively. Before eradication, the sensitivity and specificity were 96.2‒100% and 83.3‒100%, respectively, with a cutoff value of 2.5‰ [10, 11]. After eradication, the sensitivity and specificity were 66.7‒100% and 95.8‒98.8%, respectively, with a cutoff value of 3.5‰ [12]. Assessment of *H. pylori* eradication should be performed at least 4‒6 weeks after treatment. However, UBT may yield false-positive results owing to the presence of urease-positive bacteria other than *H. pylori* [10, 13]*.* The UBT may yield false-negative results during antibiotic or PPI/P-CAB use; therefore, antibiotics should be discontinued for 4 weeks and PPI/P-CAB for 2 weeks before testing [14].

### CQ1-2

**Is** ***H. pylori*** **eradication recommended based on a positive serum** ***H. pylori*** **antibody test result?**

- Starting eradication based solely on a positive serum antibody test result is not recommended, since serum antibodies do not only reflect current infection status.

(Recommendation: strong (91.3% strong agreement), evidence level B).

Comment: Accuracy of serum antibody tests depends on the *H. pylori* strain from which the antigen is extracted; thus, local validation is important [15]. In Japan, enzyme immunoassays (EIA) are widely used in serum antibody kits. Recently, many facilities have adopted the latex method, mainly *H. pylori*-latex ‘Seiken’ (Denka Co., Ltd., Tokyo, Japan). The Denka latex kit diagnoses *H. pylori* infection with a sensitivity of 86‒98.1% and specificity of 78.0‒95%, using a cutoff value of 10.0–10.8 U/mL [16]. *H. pylori* infection should be comprehensively diagnosed using endoscopic findings (such as Kyoto Classification of Gastritis) combined with tests such as serum antibodies, UBT, SAT, RUT, histopathology, culture, and NAAT [17, 18]. Serum antibody titers decrease by less than half within 6 months after *H. pylori* eradication, making them a potential indicator of successful eradication [19, 20].

### CQ1-3

**Is antibiotic susceptibility testing of** ***H. pylori*** **necessary before initiating eradication therapy?**

- Antibiotic susceptibility testing should be performed before initiating *H. pylori* eradication, and the eradication regimen expected to achieve the highest eradication rate should be selected. NAAT is also useful for detecting bacterial genetic mutations associated with clarithromycin (CAM) susceptibility.

(Recommendation: strong (77.3% strong agreement), evidence level A).

Comment: CAM resistance is a critical factor that hinders *H. pylori* eradication. In Japan, the CAM-resistance rate remains high (35.5%), although resistance to amoxicillin (AMPC) and metronidazole (MNZ) is low [21]. Japanese studies comparing individualized regimens (P-CAB, AMPC, CAM, or MNZ) based on susceptibility testing with empirical regimens (P-CAB, AMPC, and CAM) found that individualized treatment achieved significantly higher eradication rates and reduced costs [22, 23]. Traditional culture-based sensitivity testing is time-consuming; however, new molecular diagnostic methods offer advantages. NAAT can detect mutations in the CAM-resistance gene (23S rRNA) from gastric fluid within approximately 1 h with a high accuracy (97.0%), enabling rapid, tailored treatment [24]. Continued surveillance of antibiotic resistance trends is essential for optimizing eradication regimens.

### CQ1-4

**Is serum pepsinogen (PG) test useful for diagnosing** ***H. pylori*** **infection?**

- PG II levels and the PG I/II ratio are useful auxiliary diagnostic tools for *H. pylori* infection. An increase in the PG I/II ratio after treatment is useful for assessing successful *H. pylori* eradication. However, these methods do not directly detect bacteria or their components.

(Recommendation: weak (40.9% strong, 36.4% weak agreement), evidence level C).

Comment: The serum PG test is a non-invasive blood test that reflects gastric inflammation and serves as a useful auxiliary tool for diagnosing *H. pylori*. For infection diagnosis, high PG II levels (sensitivity, 86.5‒90.0%; specificity, 66.0‒90.7%) or a low PG I/II ratio (sensitivity, 86.8‒89.0%; specificity, 79.6‒89.9%) are effective indicators [25, 26]. To confirm *H. pylori* eradication, successful treatment reduces inflammation and increases the PG I/II ratio. Criteria based on the percentage increase relative to pretreatment levels demonstrate high performance (sensitivity, 87.8‒100%; specificity, 83.3‒97.2%) [27–29].

## Treatment

**Fig. 2:**
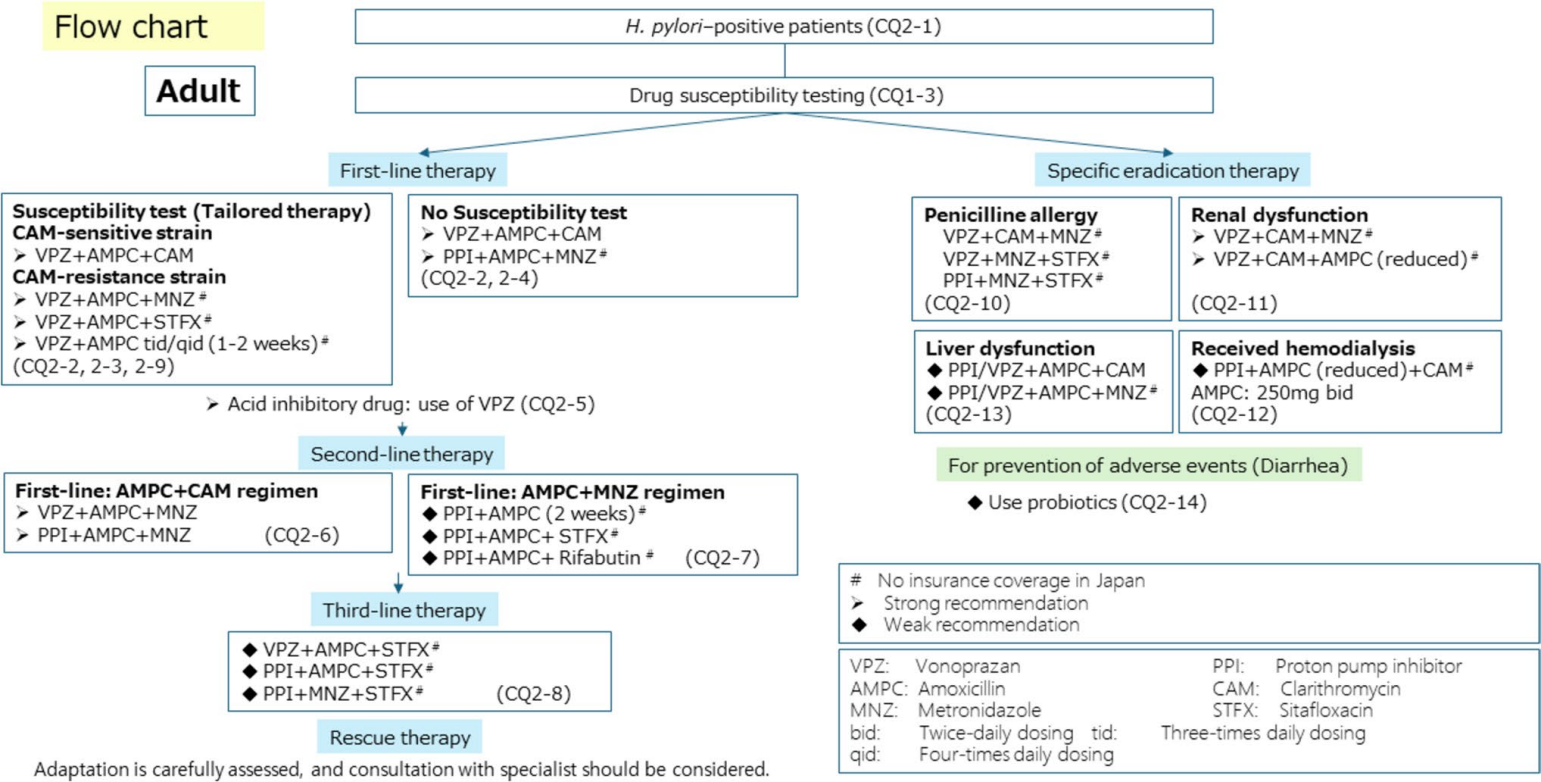
Treatment flowchart for evidence-based eradication therapy for *H. pylori* infection in Japan

Treatment flowchart for evidence-based eradication therapy for *H. pylori* infection in Japan is shown in Fig. 2. It is recommended that all infected individuals undergo eradication treatment and receive antimicrobial susceptibility testing before eradication therapy. Consultation with a specialist is essential for rescue therapies or eradication treatments in special situations. The metronidazole-based regimen is not covered by the current Japanese health insurance as the first-line eradication therapy and should be prescribed with caution for infections caused by metronidazole-resistant bacteria and because of safety concerns.

## Selected BQs in treatment

**What is the optimal dosage of CAM for first-line eradication therapy: 400 mg/day or 800 mg/day?**

- The recommended CAM dose for first-line eradication is 400 mg/day (200 mg twice daily).

**Fig. 3:**
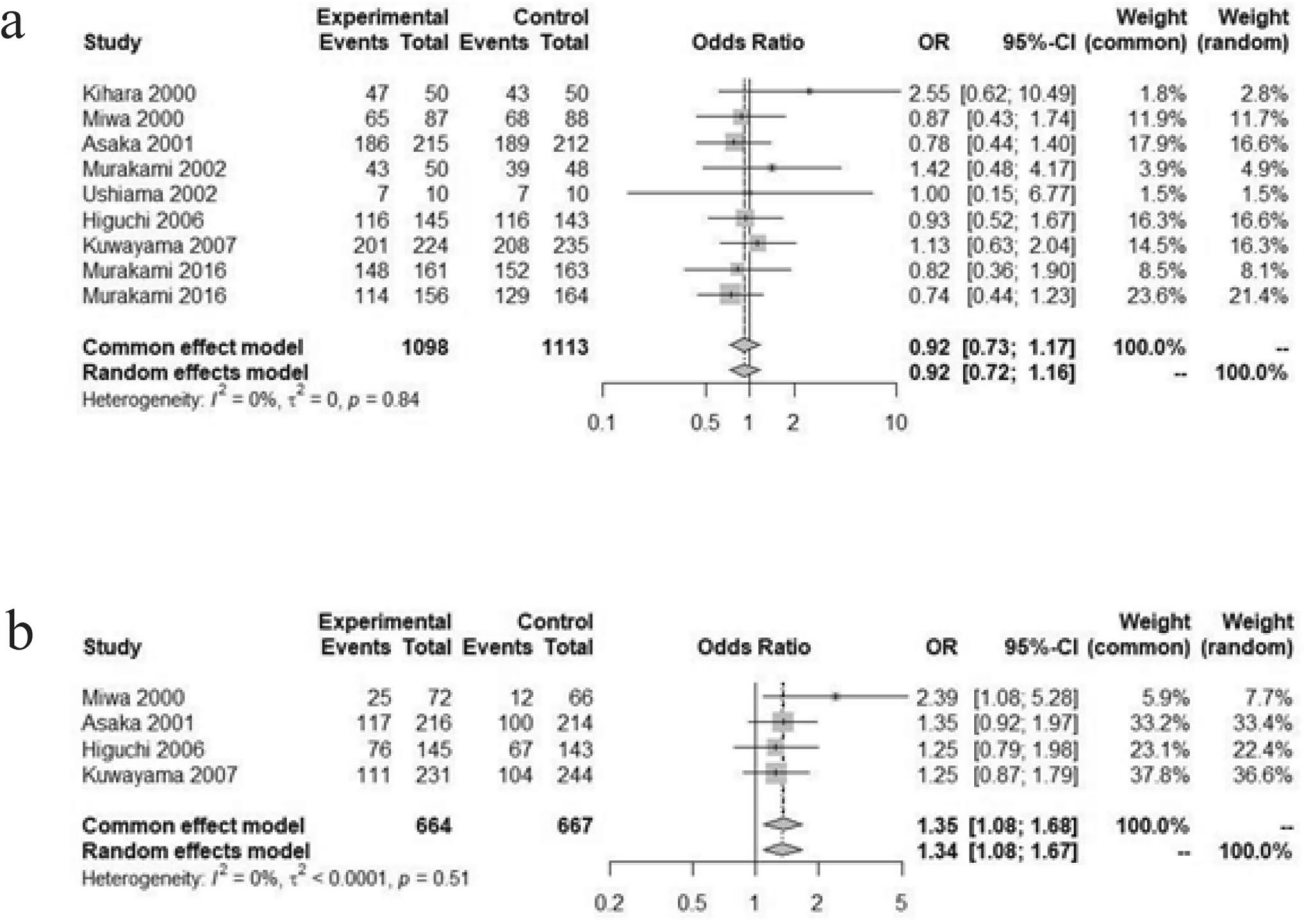
Systematic review and meta-analysis of VAC 800 versus VAC 400. **a** Efficacy: eradication rates. **b** Safety: incidence of adverse events. Study groups: Experimental group: VAC 800. Control group: VAC 400. Statistical annotations and interpretation: Effect size: Results of this systematic review and meta-analysis are expressed as odds ratios (ORs) with 95% confidence intervals (CIs). Point estimate and weight: The square in the center of each horizontal line represents the point estimate of an individual study. The size of the square indicates the weight assigned to the study, proportional to its precision and sample size. Pooled estimate: The diamond at the bottom represents the overall combined effect. In **a** (efficacy), a result to the right of the vertical line favors VAC 800. In **b** (safety), a result to the right of the vertical line indicates a higher risk of adverse events in that group. Significance: Statistical significance is achieved if the 95% CI (horizontal line or diamond) does not cross the line of no effect (vertical line at 1.0). Heterogeneity: The I^2^ statistic describes the percentage of total variation across studies due to heterogeneity, rather than chance. Common and random-effects models were used for this analysis. *VAC400* vonoprazan (VPZ) + amoxicillin (AMPC) + clarithromycin (CAM) 400 mg/day, *VAC800* VPZ + AMPC + CAM 800 mg/day

Comment: A systematic review and meta-analysis evaluated the optimal dosage of CAM in first-line 7-day triple therapy for *H. pylori* eradication, which consists of a PPI, vonoprazan (VPZ), AMPC, and CAM. It compared the efficacy and safety of 400 mg/day versus 800 mg/day of CAM [30–37]. A meta-analysis of eight randomized controlled trials (RCTs) in Japan found no significant difference in eradication rates between the two dosages (odds ratio (OR), 0.92; 95% confidence interval (CI), 0.73‒1.17). This evidence had high certainty and low heterogeneity (Fig. 3a). Regarding safety, a meta-analysis of four RCTs showed a significantly higher frequency of adverse events at 800 mg/day (OR, 1.35; 95% CI, 1.08‒1.68). Although no serious safety concerns were identified in either group, the increased incidence of side effects at higher doses was notable (Fig. 3b). In Japan, given that the 800 mg/day dose offers no superior eradication benefit and is associated with a higher frequency of adverse events, the 400 mg/day dose (200 mg twice daily) is recommended.

**What is the optimal treatment duration for** ***H. pylori*** **eradication therapy?**

- In Japan, *H. pylori* eradication therapy is recommended for 7 days.

Comment: A meta-analysis comparing 7-day and 14-day eradication regimens showed that while the 14-day regimen increased the rates of adverse events in the PPI + AMPC + CAM (PAC) regimen (16.0% vs. 19.6%, p = 0.01), it significantly improved the eradication rate (74.9% vs. 83.5%, p < 0.001) [38]. Based on this evidence, the clinical international guidelines and consensus reports for *H. pylori* infection recommend a 14-day PAC regimen over a 7-day regimen [39, 40]. In contrast, in an RCT conducted in Japan comparing 7-day and 14-day PAC regimens, no significant difference was observed between the two groups [41]. When VPZ was used, equivalent results were observed with both the 7-day VPZ + AMPC + CAM (VAC) and 14-day PAC regimens, which carry a higher risk of adverse events [42]. Therefore, VAC is the standard treatment in Japan.

### CQ2-1

**Should** ***H. pylori*** **eradication therapy be performed in elderly patients?**

- *H. pylori* eradication therapy is beneficial for older adult patients, effectively preventing short-term peptic ulcer recurrence, and showing good tolerability.

(Recommendation: weak (38.1% strong, 33.3% weak agreement), evidence level C).

Comment: Large cohort studies and randomized trials in populations aged ≥ 60 or ≥ 65 years have demonstrated significant reductions in gastric cancer incidence, especially after long-term follow-up (standardized incidence ratio, 0.42; 95% CI, 0.18–0.84) [43], as well as marked prevention of ulcer relapse [44]. In older adult aspirin users, eradication reduced the risk of ulcer bleeding within 2.5 years [45]. Adverse events occurred in only 8.5–9.7% of older adult patients, similar to the rates observed in younger adults [46]. Moreover, cost-effectiveness analyses indicate that screening and eradication in individuals aged 60–80 years yield a greater quality-adjusted life year at lower costs than no intervention [47]. Overall, *H. pylori* eradication in older adults appears to be effective, safe, and economically reasonable, although data for those aged ≥ 80 years remain limited.

### CQ2-2

**Which acid-suppressing drug is better for** ***H. pylori*** ***eradication therapy: VPZ or PPI?***

- VAC is strongly recommended as first-line therapy, regardless of CAM susceptibility. For second-line therapy, either PPI + AMPC + MNZ (PAM) or VPZ + AMPC + MNZ (VAM) is recommended.

(Recommendation: strong (90% strong agreement), evidence level A).

**Fig. 4:**
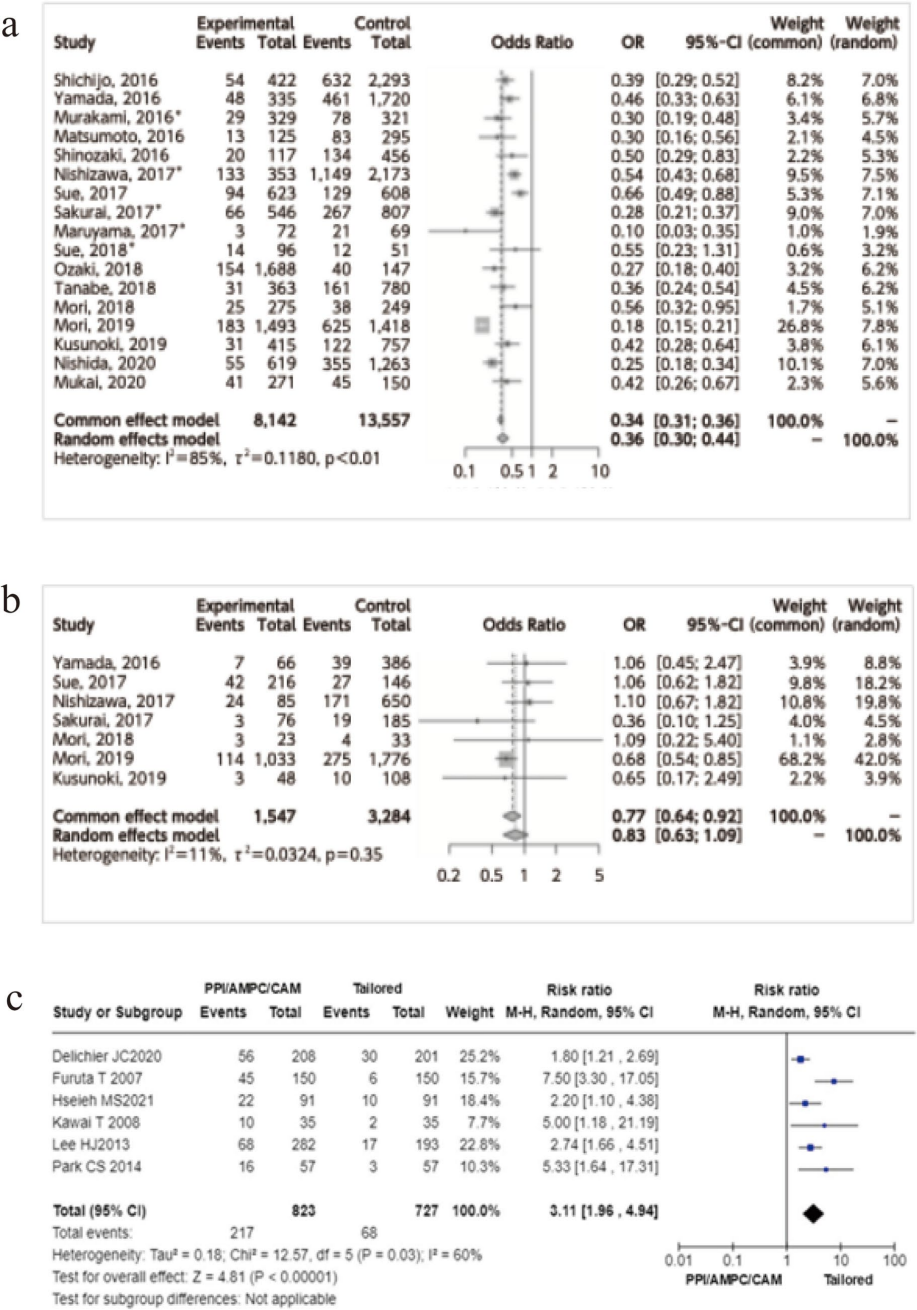
**a** Systematic review and meta-analysis of eradication rates for PAC and VAC therapies in Japan (first-line eradication regimen): ITT analysis (* indicates RCT; others indicate non-RCT). Study groups: Experimental group: VAC and Control group: PAC. **b** Systematic review and meta-analysis of eradication rates of PAM and VAM therapies in Japan (second-line eradication regimen): ITT analysis (non-RCT). Study groups: Experimental group: VAM and Control group: PAM. In both (**a**) and (**b**), events refer to the number of patients with eradication failure. Total refers to the total number of enrolled patients. Statistical annotations and interpretation: Effect size: Results of this systematic review and meta-analysis are expressed as odds ratios (ORs) with 95% confidence intervals (95% CI). Point estimate and weight: The square in the center of each horizontal line represents the point estimate of an individual study. The size of square indicates the weight assigned to the study, proportional to its precision and sample size. Pooled estimate: The diamond at the bottom represents the overall combined effect. In **a**, a result to the left of the vertical line favors VAC, and in **b**, a result to the left of the vertical line favors VAM with no significance. Significance: Statistical significance is achieved if the 95% CI (horizontal line or diamond does not cross the line of no effect (vertical line at 1.0). Heterogeneity: The I^2^ statistic describes the percentage of total variation across studies due to heterogeneity, rather than chance. Common and random-effect models were used for the analysis. *PPI* proton-pump inhibitor, *PAC* PPI + AMPC + CAM, *VAC* VPZ + AMPC + CAM, *PAM* PPI + AMPC + MNZ, *VAM* VPZ + AMPC + MNZ, *ITT* intention-to-treat, *RCT* randomized controlled trial

Comment: VPZ-based first-line regimens demonstrated significantly higher eradication rates than PPI-based regimens (intent-to-treat, ITT analysis: 87.6% vs. 70.8%), as shown in the meta-analyses in Fig. 4a. This advantage was particularly pronounced in CAM-resistant strains (75% vs. 38%). For second-line therapy, the success rates were comparable between the VPZ- and PPI-based regimens, as shown in the meta-analyses in Fig. 4b. Side effect rates do not differ significantly between the regimens [37, 48–50]. VPZ-based regimens can be more cost-effective because they reduce the need for second-line treatments [51].

Recommended regimens

- First-line eradication: VAC (20 mg/750 mg/200 mg) bid for 1 week
- Second-line eradication: VAM (20 mg/750 mg/250 mg) bid for 1 week or PAM (standard dose*/750 mg/250 mg) bid for 1 week

*: esomeprazole 20 mg, rabeprazole 10 mg, or lansoprazole 30 mg.

## CQ2-3

**In first-line eradication therapy, which is more useful: routine insurance-covered standard triple therapy or tailored eradication?**

- For first-line *H. pylori* eradication, tailored eradication therapy based on antimicrobial susceptibility testing yields higher eradication rates than standard triple therapy and is, therefore, recommended. As adverse events and drug–drug interactions can occur, patient-specific selection of agents is advised.

(Recommendation: strong (100% strong agreement), evidence level A).

Comment: A meta-analysis of RCTs comparing PAC with susceptibility-guided regimens demonstrated the superiority of tailored eradication (risk ratio (RR), 3.11; 95% CI, 1.96–4.94) (Fig. 4c). In Japan, VAC is standard and effective against CAM-susceptible strains but is less effective against resistant strains. A nationwide cohort study reported eradication rates of 96.3% in CAM-susceptible and 82.9% in resistant strains, and substituting CAM with MNZ achieved 98.0% eradication [52]. A single-center study similarly showed 94.0% eradication with standard therapy versus 98.5% with MNZ-based therapy [23]. These findings confirm the benefits of susceptibility-guided therapies. Personalization should also consider comorbidities and cytochrome P450-mediated interactions. Tailored eradication in the first-line stage maximizes both efficacy and safety.

### CQ2-4

**What regimen should be selected when antimicrobial susceptibility testing is performed prior to first-line therapy?**

- If the strain is susceptible to CAM, VAC is recommended. In cases of CAM resistance, one of the following regimens should be selected based on susceptibility testing for MNZ and fluoroquinolones: VAM, VPZ + AMPC + sitafloxacin (STFX), VAS for 1 week, or VPZ + AMPC (VA) for 2 weeks.

(Recommendation: strong (100% strong agreement), evidence level B).

Comment: Antimicrobial susceptibility testing-guided eradication regimens achieve significantly higher success rates than standard triple therapy [53–58]. Susceptibility testing was conducted using 23S rRNA and gyrA mutation detection or agar dilution methods. VPZ is known to be superior to PPIs as an acid suppressant. STFX is preferred over levofloxacin (LVFX).

Recommended regimens.

•CAM-susceptible: VAC (20 mg/750 mg/200 mg) bid for 1 week
•CAM-resistant, MNZ-susceptible: VAM (20 mg/750 mg/250 mg) bid for 1 week
•CAM-resistant, MNZ-unknown, and quinolone-susceptible strains: VAS (20 mg/750 mg/100 mg) bid for 1 week
- CAM-resistant, MNZ-unknown, and quinolone-resistant/unknown: VAM or VA (20 mg bid/500 mg qid) for 2 weeks
- CAM-resistant, MNZ-resistant, and quinolone-susceptible: VAS (20 mg/750 mg/100 mg) bid for 1 week
- CAM-resistant, MNZ-resistant, and quinolone-unknown: VAS (20 mg/750 mg/100 mg) bid for 1 week or VA (20 mg bid/500 mg qid) for 2 weeks
- CAM-resistant, MNZ-resistant-, and quinolone-resistant: VA (20 mg bid/500 mg qid) for 2 weeks

### CQ2-5

**Which eradication therapy should be selected if susceptibility testing is not performed at first-line therapy?**

•The recommended eradication regimens are either VAC or PAM.

(Recommendation: strong (90.5% strong agreement), evidence level A).

**Fig. 5:**
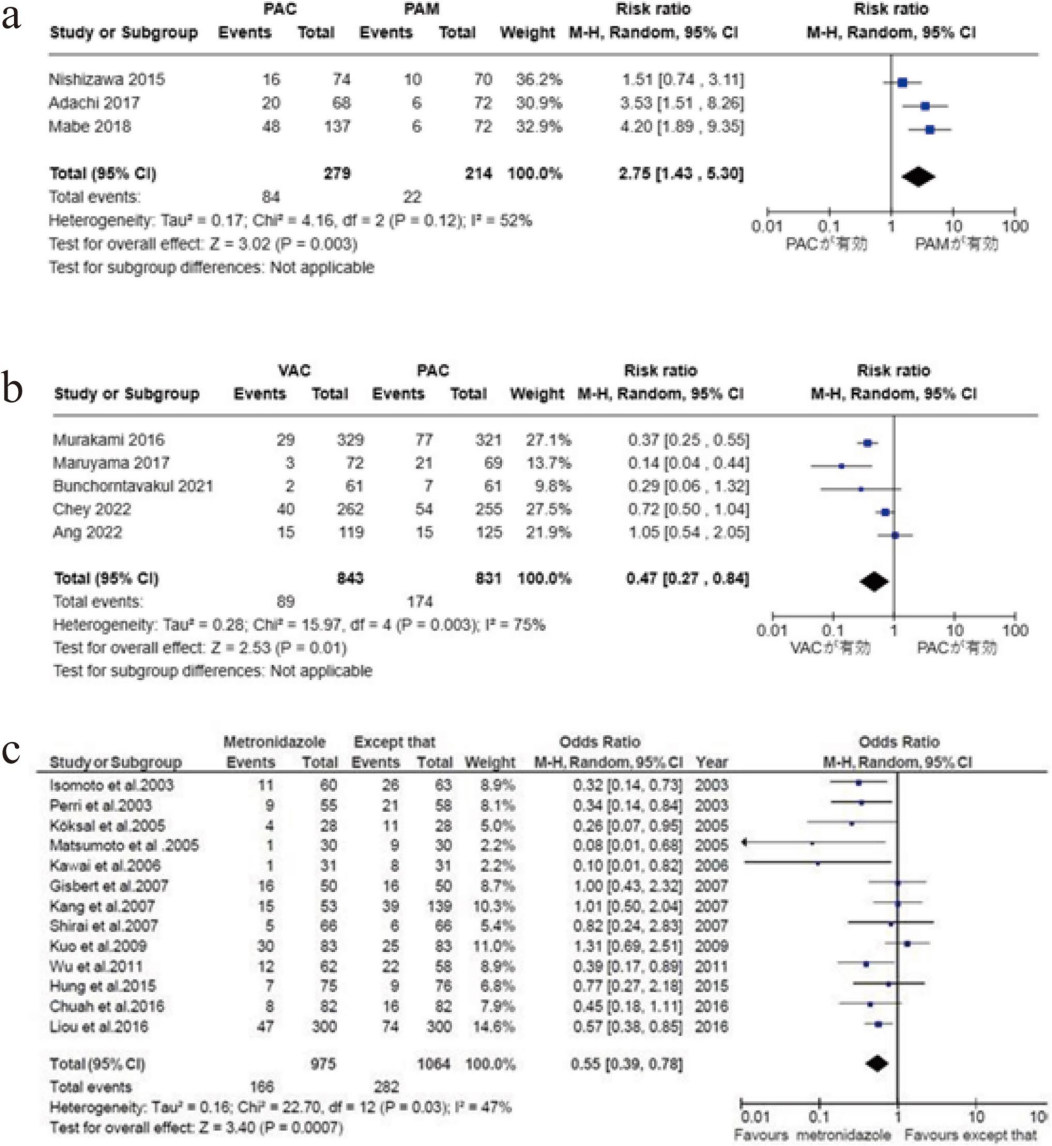
Forest plots of eradication rates between PAM and PAC therapy in Japan (**a**) and eradication rates (ITT analysis) between VAC and PAC in randomized control trials (**b**)

Comment: The prevalence of CAM-resistant strains in Japan currently exceeds 30% [21]. For patients living in areas with high CAM-resistance rates (> 15%), the Maastricht VI/Florence Consensus Report suggests that first-line therapy using CAM should not be selected when susceptibility testing is not performed before eradication [39]. In Japan, with low MNZ- and high CAM-resistance rates (approximately 5% and > 30%, respectively), the PAM regimen shows a higher eradication rate than the PAC regimen in meta-analyses (Fig. 5a) [59]. However, evidence of VAM efficacy is insufficient. Given that the degree and duration of acid inhibition are related to eradication rates [60], the VAC regimen demonstrates higher efficacy than the PAC regimen (Fig. 5b).

Recommended regimens.

- VAC (20 mg/750 mg/200 mg) bid for 1 week
- PAM (standard dose/750 mg/250 mg) bid for 1 week

### CQ2-6

**What is the recommended second-line eradication regimen when AMPC and CAM are selected for first-line therapy?**

- Either 7-day VAM or PAM regimen is recommended.

(Recommendation: strong (85.0% strong agreement), evidence level A).

Comment: Regional variations in antibiotic resistance profiles necessitate tailored *H. pylori* eradication strategies [61, 62]. Globally, high MNZ (> 30%) and moderate CAM (10–20%) resistance often favors bismuth- or fluoroquinolone-based second-line therapies. However, in Japan, the pattern is unique, with high CAM resistance (30–40%) but exceptionally low MNZ resistance (< 5%) [61, 62]. Consequently, MNZ-containing regimens are the standard second-line therapy. A meta-analysis confirmed that MNZ-containing regimens had significantly lower failure rates (OR, 0.55; 95% CI, 0.39–0.78) than MNZ-free regimens, proving superior in both low- (OR 0.29) and high-resistance (OR 0.66) regions (Fig. 5c) [63]. Many Japanese studies have reported high eradication rates (ITT > 80%, per-protocol, PP > 90%) [64]. VPZ further enhanced these results [33, 63], highlighting regional optimization.

Recommended regimens.

- VAM (20 mg/750 mg/250 mg) bid for 1 week
- PAM (standard dose/750 mg/250 mg) bid for 1 week

### CQ2-7

**What is the recommended rescue therapy for CAM-resistant patients failing first-line VPZ/PPI + AMPC + MNZ?**

- Potential therapeutic options include PPI + AMPC (PA), either as 14-day dual therapy or as triple therapy combined with rifabutin (RFB) or a quinolone.

(Recommendation: weak (42.1% strong, 52.6% weak agreement), evidence level B).

Comment: CAM-resistant patients who fail first-line MNZ-based therapy are presumed to have dual (CAM/MNZ) resistance. As no studies exist specifically for such populations, evidence was extrapolated from RCTs and subgroup analyses of patients with confirmed dual resistance. Among primary RCTs, a 14-day furazolidone/bismuth quadruple therapy showed the highest efficacy (95.2%) [65]. However, these drugs have not been approved in Japan. Other regimens with moderate efficacy include 14-day PA high-dose dual therapy [66, 67] and 7-day RFB-based triple therapy (PPI + AMPC + RFB [PAR]) [67]. A subgroup analysis also reported the high efficacy of a 14-day quinolone-based triple therapy (PPI + AMPC + LVFX; PAL) [68]. The PPI+AMPC+quinolone triple therapy showed acceptable safety profiles, as confirmed by Japanese trials [69, 70].

Recommended regimens:

•High-dose PA (AMPC 3 g/day) for 2 weeks
•PAR (AMPC 2 g, REB 300 mg/day) for 1 week
•PAL (AMPC 2 g, LVFX 500 mg/day) for 2 weeks

### CQ2-8

**Recommended third-line eradication therapy in Japan?**

- A 7-day VAS triple therapy is preferred, anticipating the highest eradication rate and acceptable safety. Alternative regimens include PAS, PPI + MNZ + STFX (PMS), and VPZ + AMPC + RFB (VAR).

(Recommendation: weak (36.8% strong, 63.2% weak agreement), evidence level A).

**Fig. 6:**
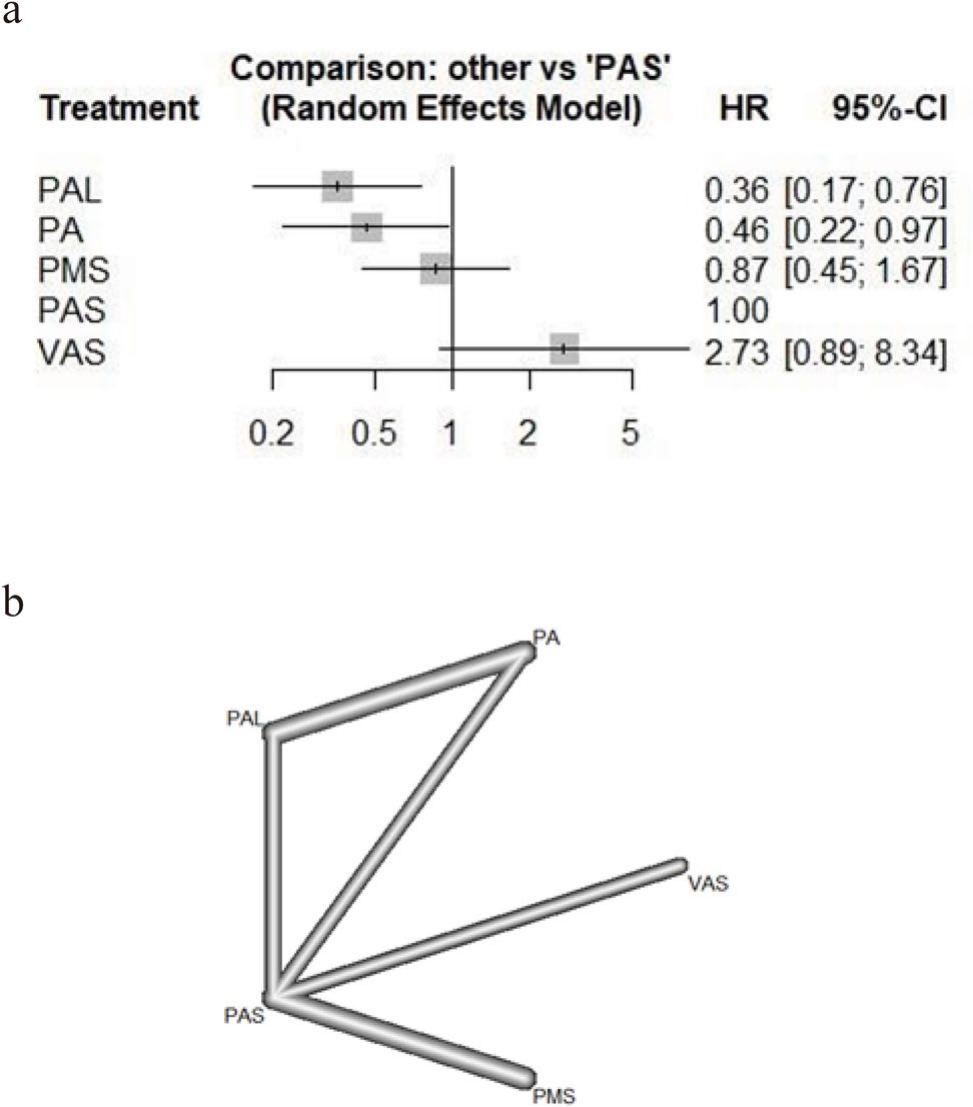
Forest plot of the OR with 95% CI for MNZ-containing versus MNZ-free regimens: **a** effect size for each treatment (random-effects model); **b** network plot. *PA* PPI + AMPC dual therapy, *PAL* PPI + AMPC + LVFX triple therapy, *PAS* PPI + AMPC + STFX triple therapy, *PMS* PPI + MNZ + STFX triple therapy, *VAS* VPZ + AMPC + STFX triple therapy. *LVFX* levofloxacin, *STFX* sitafloxacin, *OR* odds ratio, *CI* confidence interval

Comment: CQ2-8 addresses the optimal third-line *H. pylori* therapy in Japan following the failure of standard 7-day first-line (CAM-based) and second-line (MNZ-based) triple therapies. A systematic review (17 studies: 6 RCTs and 11 single-arm studies) and network meta-analysis (NMA) of five RCTs were conducted (Fig. 6a). The NMA found that 7-day VAS likely yielded the highest eradication rate (RR, 2.73; 95% CI, 0.89–8.34) compared to PPI-based alternatives (Fig. 6b). However, the evidence quality was moderate owing to imprecision (CI crossed 1.0) [69–74]. Single-arm studies support this regimen (approximately 90% efficacy). Although most therapies have reported no major safety issues, PAR was terminated early because of its poor tolerability. VAR also demonstrated promising efficacy (> 90%).

Recommended regimen: VAS (20 mg/750 mg/100 mg) bid for 1 week.

### CQ2-9

**When should dual therapy with VA be selected?**

- In first-line eradication therapy, we recommend dual therapy with the VA regimen when *H. pylori* is resistant to CAM or when there is a risk of disadvantages related to the second antimicrobial agent, such as CAM, MNZ, and STFX.

(Recommendation: weak (36.8% strong, 63.2% weak agreement), evidence level A).

Comment: A meta-analysis showed no significant difference in eradication rates between VA and VAC (pooled RR, 0.98; 95% CI, 0.95‒1.01; p = 0.100) [49, 75–79]. However, the VAC eradication rate for CAM-susceptible strains was higher than that for VA [78]. In two Japanese studies, eradication rates exceeded 90% when VPZ 20 mg bid and 500 mg of AMPC were administered tid or qid [75, 80].

### CQ2-10

**What eradication regimen should be selected for patients with penicillin allergy?**

- For patients with penicillin allergy, *H. pylori* eradication should be guided by antimicrobial susceptibility testing whenever possible. Suitable regimens include VPZ + CAM + MNZ (VCM), VPZ + MNZ + STFX (VMS), or PPI + MNZ + STFX (PMS).

(Recommendation: none).

Comment: Internationally, bismuth-containing quadruple therapy is widely recommended and achieves high eradication rates in patients with penicillin allergy. In Japan, a prospective study reported 100% eradication with a VPZ-based regimen and VCM compared with 83.3% with a PPI-based regimen and PCM [81], with consistent findings in retrospective cohorts [82, 83]. Rescue regimens using VMS/PMS achieved eradication rates of 88.2–100% [82, 84]. However, efficacy is markedly reduced in strains resistant to both MNZ and STFX [85]. Therefore, susceptibility-guided antibiotic selection is recommended. Although the above regimens appear feasible, the current evidence remains insufficient.

Recommended regimens.

- VPZ (20 mg bid), CAM (200/400 mg bid), and MNZ (250 mg bid) for 1 week
- VPZ (20 mg bid), MNZ (250 mg bid), and STFX (100 mg bid) for 1 week.
- PPI, standard dose bid, MNZ (250 mg bid), and STFX (100 mg bid) for 1 week.

### CQ2-11

**What regimen should be selected for patients with impaired renal function?**

- In patients with impaired renal function, VCM—administered with an appropriate dose reduction of antibiotics based on renal function—is recommended, whereas VAC is considered the next alternative. However, the indications for treatment should be carefully considered.

(Recommendation: strong (84.2% strong agreement), evidence level C).

Comment: According to an RCT comparing the effects of PCM and PAC in patients with impaired renal function, PCM was superior to PAC in terms of the incidence of acute renal failure and eradication rates [86]. The recommended regimens for patients with impaired renal function include VPZ, CAM, and MNZ [87]. Three studies evaluating the effects of PAC therapy in patients with and without chronic kidney disease demonstrated similar eradication rates and adverse effects in both groups [88–90]. VAC at reduced doses, depending on renal function, remains a potential option.

### CQ2-12

**Which eradication therapy should be selected in patients receiving hemodialysis?**

- For patients receiving hemodialysis, the AMPC dose should be reduced to 250 mg (one-third dose), and the recommended regimen is PAC.

(Recommendation: weak (58.0% strong, 42.0% weak agreement), evidence level C).

Comment: Given the delayed pharmacokinetics of AMPC, adverse effects should be minimized [91]. A recent meta-analysis demonstrated comparable eradication rates between patients and healthy individuals, both with the same and reduced dosage regimens (RR, 0.85; 95% CI, 0.48–1.50) [92]. The adverse event rates in the reduced-dose regimen were similar to those in healthy individuals (1.26 [0.23–6.99]) [92]. From a pharmacological perspective, the regimen should consider the dosage (1/2 to 1/3), dosing frequency (bid), and dosing timing (after hemodialysis) [92]. There is no evidence of VPZ-based eradication regimens in patients undergoing hemodialysis.

Recommended regimen.

- PPI (standard dose bid) + AMPC (250 mg bid) + CAM (200 mg bid) for 1 week.

*Hemodialysis in the morning: On hemodialysis days, medications after hemodialysis ends and with dinner.

*Hemodialysis in the afternoon or evening: On hemodialysis days, medications should be administered after breakfast before hemodialysis.

### CQ2-13

**What is the recommended eradication regimen for patients with hepatic impairment?**

- Standard regimens are options for hepatic impairment; however, indications require careful assessment of the severity and eradication of hepatic dysfunction. Adverse events should be closely monitored during treatment.

(Recommendation: weak (35.0% strong, 65.0% weak agreement), evidence level B).

Comment: Most *H. pylori* eradication drugs are metabolized in the liver, and hepatic dysfunction may affect treatment outcomes. Although randomized controlled trials are lacking, observational studies have reported comparable efficacy and safety in patients with liver disease and those with normal hepatic function. Eradication rates remained high in patients with cirrhosis/chronic hepatitis and nonalcoholic fatty liver disease/nonalcoholic steatohepatitis, without significant adverse events [93–97]. Pharmacokinetic studies have shown reduced clarithromycin metabolism in patients with severe hepatic dysfunction, but without clinically relevant effects on clearance or safety. Therefore, standard regimens are generally applicable with careful assessment and monitoring.

Recommended regimens.

- VPZ (20 mg bid) or EPZ (20 mg bid) + AMPC (750 mg bid) and CAM (200 mg bid) for 1 week
- VPZ (20 mg bid) or EPZ (20 mg bid) + AMPC (750 mg bid) and MNZ (250 mg bid) for 1 week

### CQ2-14

**Should probiotics be used concomitantly during eradication therapy?**

- Co-administration of probiotics prevents side effects.

(Recommendation: weak (20.0% strong, 75.0% weak agreement), evidence level C).

Comment: A review of 17 international RCTs found that adding probiotics reduced side effects, such as diarrhea and abdominal pain, in 13 studies. However, a significant improvement in the eradication rate was observed in only 3 of the 17 RCTs [98]. A Japanese study reported that adding probiotics to a PPI-based regimen improved the success rate, whereas another study suggested that probiotics may decrease the effectiveness of VPZ-based therapy [99].

## Gastric cancer prevention (adults)

### CQ3-1

**Does serum anti-*H. pylori*** **antibody and/or PG tests in asymptomatic individuals contribute to gastric cancer prevention?**

The evidence is insufficient to recommend serum-based testing alone for gastric cancer prevention; any benefit depends on appropriate post-test management. The guideline committee members did not vote.

Comment: Risk-stratified screening, which combines serum testing, eradication, and tailored endoscopic surveillance, is reportedly useful for gastric cancer prevention [100, 101]. In Japan, serum anti-*H. pylori* antibody and PG tests are widely used in screening and health check-ups; however, organized programs generally include testing and result notification only, without eradication therapy or endoscopic follow-up. Limited data on post-test management raise clinically relevant questions about whether testing alone contributes to gastric cancer prevention. Accordingly, CQ3-1 evaluated serum testing alone as the intervention and found insufficient evidence of a preventive effect on gastric cancer outcomes. As *H. pylori* prevalence declines, large interventional trials are unlikely to be conducted, making high-quality observational data with appropriate follow-up increasingly important for evaluating risk-stratified gastric cancer prevention strategies.

### CQ3-2

**Are annual serum anti-*H. pylori*** **antibody and/or PG tests necessary for gastric cancer prevention?**

- Annual testing is not recommended for gastric cancer prevention.

(Recommendation: strong (85.0% strong agreement), evidence level C).

Comment: Although antibody titers increase with infection and decline following eradication, post-eradication antibody changes do not predict gastric cancer risk [102–105]. PG I/II ratios vary with age and infection status [106]. However, no evidence supports that repeated testing contributes to cancer prevention. Therefore, clear evidence supporting annual serum H. pylori antibody or PG testing, including ABC screening, for gastric cancer prevention, is lacking. Testing is reasonable when an infection is clinically suspected; however, routine annual screening is not justified.

### CQ3-3

**Does gastric cancer screening (radiography or endoscopy) in the general population contribute to diagnosis and primary prevention?**

•Gastric cancer screening can detect *H. pylori* infection and may contribute to the primary prevention of gastric cancer. However, further studies are required, and no formal recommendations can be made. The guideline committee members did not vote.

Comment: Gastric cancer screening using radiography or endoscopy is officially implemented only in Japan and South Korea [107], and these imaging modalities are useful for diagnosing *H. pylori*-associated gastritis [108]. Since most gastric cancers in Japan arise from *H. pylori*-infected gastritis [109], national health insurance coverage for eradication therapy was expanded in 2013. The primary preventive role of gastric cancer screening is to identify individuals with *H. pylori*-infected gastritis and refer them for eradication therapy. Most infected individuals are asymptomatic [110] and do not undergo routine testing and treatment. Therefore, screening healthy individuals offers a valuable opportunity to detect *H. pylori* infections. Although screening is expected to contribute to primary prevention through eradication, no clinical study has confirmed this benefit.

### CQ3-4

**What is the optimal timing for** ***H. pylori*** **eradication for gastric cancer prevention in adults?**

- *H. pylori* eradication therapy for gastric cancer prevention is strongly recommended at an early age.

(Recommendation: strong (95% strong agreement), evidence level C).

Comment: Early eradication of *H. pylori* markedly reduces the long-term risk of gastric cancer. The Kyoto Global Consensus [111] and Maastricht VI/Florence Consensus [39] emphasize that eradication before the onset of atrophic gastritis or intestinal metaplasia provides the greatest preventive benefits. Quantitative evidence supports this recommendation: a Korean nationwide cohort study [112] reported hazard ratios (HR) of 0.34 (< 45 years), 0.38 (45–49 years), 0.57 (50–54 years), and 0.62 (55–59 years) for gastric cancer compared with eradication at ≥ 75 years (*p* < 0.001). In Hong Kong, eradication at ≥ 60 years reduced incidence by 18% [43]. A Taiwanese meta-analysis showed a 3% decrease in gastric cancer risk for every 1-year earlier eradication [113]. A cohort of U.S. veterans reported cumulative cancer incidences of 0.37%, 0.50%, and 0.65% at 5, 10, and 20 years, respectively, after infection diagnosis [114]. A post hoc analysis of an RCT conducted in China demonstrated that eradication significantly reduced gastric cancer incidence (OR: 0.36) and mortality (HR: 0.26) at 15 years, particularly in patients aged ≥ 55 years or with intestinal metaplasia [115]. In summary, multiple large cohort studies have indicated that *H. pylori* eradication is most beneficial when performed early, ideally before histologic atrophy develops [116]. However, randomized trials comparing early versus delayed eradication are lacking.

### CQ3-5

**Is it necessary to repeat diagnostic tests to confirm** ***H. pylori*** **eradication?**

- Repeating diagnostic tests to confirm *H. pylori* eradication is generally not necessary. However, if persistent infection or reinfection is clinically suspected, repeated diagnostic tests are recommended.

(Recommendation: strong (100% strong agreement), evidence level C).

Comment: In Japan, the assessment of *H. pylori* eradication results is recommended at least 4 weeks after the completion of therapy using either UBT or SAT with monoclonal antibodies. Although both methods have high sensitivity and specificity, false-positive and false-negative results may occur [117]. Reinfection can occur after successful eradication [118, 119]. Confirmation of successful *H. pylori* eradication should be rigorous to minimize false-negative results. Routine repetition of tests is not economically viable. However, when persistent *H. pylori* infection or reinfection is suspected based on the clinical course or endoscopic findings, repeated testing should be performed.

### CQ3-6

**Which patients require long-term follow-up after eradication?**

- Since it is currently impossible to identify a group of patients for whom surveillance is unnecessary, long-term surveillance is recommended for post-eradication cases.

(Recommendation: strong (100% strong agreement), evidence level C).

Comment: A systematic review was conducted to address this question, but two studies [120, 121] compared outcomes between patients with and without regular testing after *H. pylori* eradication. Neither study used a control group to examine the factors determining the need for follow-up after eradication. Therefore, it is currently impossible to identify patient groups in whom surveillance is unnecessary. To implement surveillance efficiently, surveillance intervals should be individually set by evaluating risk factors such as severe atrophy, intestinal metaplasia, and history of gastric cancer.

## Gastric cancer prevention (adolescents)

There is no insurance coverage for test-and-treat and eradication therapy in children, and the metronidazole-based regimen is not covered by the current Japanese health insurance as the first-line eradication therapy and should be prescribed with caution for infections caused by metronidazole-resistant bacteria and because of safety concerns.

### CQ4-1

**Is it recommended to perform** ***H. pylori*** **infection testing on asymptomatic adolescents?**

- We recommend implementing *H. pylori* infection testing as a public health measure for asymptomatic adolescents aged ≥ 12 years.

(Recommendation: weak (15.0% strong, 70.0% weak agreement), evidence level C).

Comment: *H. pylori* infection rate among adolescents aged ≥ 12 years is lower than that among adults (2.6‒4.8%) [122–124]. Nevertheless, considering the reduction in future gastric cancer risk through eradication before atrophy progresses, the high screening participation rate achieved by targeting groups during compulsory education, and the absence of reported harmful effects, testing for infection among asymptomatic minors may be considered meaningful. There has been prior literature on the cost-effectiveness of mass screening [125], whereas a literature search via systematic review did not identify any studies comparing the implementation of *H. pylori* infection testing with its non-implementation in asymptomatic minors. In regions where testing has already been introduced, high acceptability has been reported among the examinees.

### CQ4-2

**Is** ***H. pylori*** **eradication recommended for gastric cancer prevention in adolescents?**

- *H. pylori* eradication is recommended for adolescents with asymptomatic infections to prevent gastric cancer.

(Recommendation: weak (23.8% strong, 47.6% weak recommendation), evidence level C).

Comment: Screening and eradication of *H. pylori* during adolescence have been implemented in several Japanese municipalities as a primary strategy for gastric cancer prevention. The 2016 Japanese guidelines introduced a “gastric cancer prevention” proposal, recommending *H. pylori* testing and eradication in junior high school students. The 2014 WHO/IARC report and the 2020 Taipei global consensus also advocated early eradication in high-risk regions, highlighting the greater preventive effects in younger populations [126, 127]. *H. pylori* infection acquired in childhood induces chronic gastritis, which increases the risk of gastric cancer through progressive mucosal atrophy [128, 129]. Early eradication improves gastritis and significantly reduces cancer incidence and mortality [130]. In high-risk areas, eradication of asymptomatic children offers a favorable benefit–risk balance [131]. Early eradication before significant atrophic changes occur provides the greatest cancer preventive effect. In addition, adolescent eradication can interrupt intra-familial transmission, particularly from parents to their children. Population-based eradication in adolescents, supported by local governments, may substantially reduce the future incidence and mortality rates of gastric cancer in Japan.

### CQ4-3

**What is the recommended timing for** ***H. pylori*** **test-and-treat screening in children and adolescents?**

- *H. pylori* testing is recommended for junior high school students. Testing in high schools may also be acceptable, depending on local circumstances.

(Recommendation: weak (30.0% strong, 70.0% weak agreement); evidence level C).

Comment: No prospective or randomized studies have determined the optimal timing for non-invasive *H. pylori* testing and treatment strategies in children. Observational data from Japan have shown no clear age-related differences in *H. pylori* positivity among asymptomatic students. Screening is usually performed from the second year of junior high school through high school because serum antibody testing shows low sensitivity (51.4%) in children < 10 years [132]. Testing in junior high school ensures equity in compulsory education, allows parental involvement, and offers logistical benefits. High school testing facilitates access to reimbursed eradication therapy for individuals aged ≥ 15 years. Further studies should assess the test accuracy in children aged ≥ 10 years and consider equity, accessibility, and feasibility when defining the most appropriate screening age.

### CQ4-4

**Which diagnostic tests are recommended for** ***H. pylori*** **infection screening for test-and-treat in children and adolescents?**

- We recommend SAT or urine antibody tests.

(Recommendation: strong (75% strong agreement); evidence level C).

Comments: To date, no prospective or randomized studies have evaluated optimal screening methods for children and adolescents. Observational studies report SAT with 96.1–97% sensitivity and 94.7–98.5% specificity [133, 134]; urine antibody tests, 78.4–97.6% and 96.5–100% [135–137]; serum antibody tests, 48–100% and 33–99.5%; and UBT, 75–100% and 89–100%. As screening tests are applied to asymptomatic individuals, minimally invasive procedures are preferred. Considering the convenience of specimen collection and testing costs, stool antigen or urine antibody tests are recommended for screening.

### CQ4-5

**What are the recommended** ***H. pylori*** **eradication therapies for adolescents?**

- A 7-day PAM is recommended because of reliable RCT evidence and high eradication rates. VAC is also recommended. If antimicrobial susceptibility test is performed, PAC may also be an option.

(Recommendation: weak (5.6% strong, 83.3% weak agreement); evidence level C).

Comment: CAM-resistance rates among adolescents are high (43.4% [138], 71.1% [139]), which can cause treatment failure. As testing and treatment do not involve resistance testing, selecting an effective regimen is important. An RCT [140] reported eradication rates of 98.3% and 100% for ITT and PP analyses, respectively, for PAM, compared with 60.5% and 63.4% for PAC. Although no quantitatively comparable studies have directly compared VPZ and PPI regimens, a cohort study in the same region compared PAC (2014) with VAC (2015–2017). The ITT eradication rates were 43.5% and 82.6% for PAC and VAC, respectively [123]. VAC demonstrated a relatively high eradication rate with few adverse events. However, direct comparisons are lacking.

## Appendix

The members of the Guidelines Committee who created and evaluated the JSHR ‘Guidelines for the Management of *Helicobacter pylori* Infection in Japan: 2024 Revised Edition’ are listed below.

**Executive committee**

Chair: Tadashi Shimoyama (Aomori General Health Examination Center), Vice-Chair: Masanori Ito (Department of General Internal Medicine, Hiroshima University Hospital). Members: Takako Osaki (Department of Infectious Diseases, Kyorin University School of Medicine), Osamu Toyoshima (Gastroenterology, Toyoshima Endoscopy Clinic), Juntaro Matsuzaki (Keio University Faculty of Pharmacy), Ota Hiroyoshi (Department of Clinical Laboratory Sciences, Shinshu University School of Medicine), Mitsushige Sugimoto (Division of Genome-Wide Infectious Microbiology, Research Center for GLOBAL and LOCAL Infectious Disease, Oita University), Soichiro Sue (Department of Gastroenterology, Yokohama City University Graduate School of Medicine, Yokohama, Japan), Koji Otani (Department of Gastroenterology, Osaka Metropolitan University Graduate School of Medicine), Toshihiro Nishizawa (Department of Gastroenterology and Hepatology, International University of Health and Welfare Narita Hospital, Osamu Handa (Department of Gastroenterology and Hepatology, Kawasaki Medical School), Takahisa Furuta (Furuta Clinic for Internal Medicine), Hideki Mori (Department of Pediatrics, Hyogo Medical University), Kazuhiko Inoue (Junpukai Health Maintenance Center), Kyoko Sakai (Department of Laboratory Medicine, Suita Saiseikai Hospital/ Department of Health Informatics, School of Public Health, Kyoto University), Hajime Isomoto (Department of Gastroenterology and Nephrology, Tottori University School of Medicine), Hidekazu Suzuki (Division of Gastroenterology and Hepatology, Department of Internal Medicine, Tokai University School of Medicine), Yuji Nadatani (Department of Advanced Preventive Medicine, Osaka Metropolitan University Graduate School of Medicine), Masumi Okuda (Department of Pediatrics, Hyogo Medical University), Tamaki Ikuse (Department of Pediatrics, Juntendo University Faculty of Medicine), Toshihiko Kakiuchi (Department of Pediatrics, Faculty of Medicine, Saga University), Momoko Tsuda (Department of Gastroenterology, Sapporo Cancer Screening Center, Public Interest Foundation Hokkaido Cancer Society), Katsuhiro Mabe (Mabe Goryokaku Gastrointestinal Endoscopy Clinic), and Yasuhiro Mizuno (Maru Clinic Yokosuka).

**Evaluation committee**

Chair: Yoshihiro Fukuda (Daiichi Kyoritsu Hospital). Members: Daizo Saito (Nihonbashi Daizo Clinic) and Toshio Fujioka (Department of Gastroenterology, Oita University School of Medicine).

**Review, BQ, and FRQ writing**

Manami Inoue (National Cancer Center Institute for Cancer Control), Shogo Kikuchi (Department of Public Health, Aichi Medical University School of Medicine), Hitoshi Tsugawa (Division of Host Defense Mechanism, Tokai University School of Medicine), Mototsugu Kato (Public Interest Foundation Hokkaido Cancer Society), Tadayoshi Okimoto (Department of Gastroenterology, Oita Prefectural Hospital), Tatsushi Omatsu (Department of Gastroenterology, North Medical Center, Kyoto Prefectural University of Medicine), Tomoari Kamada (Department of Health Care Medicine, Kawasaki Medical School), Satoki Shichijo (Department of Gastrointestinal Oncology, Osaka International Cancer Institute), Akifumi Tanaka (Preventive Medicine Center, Kyorin University School of Medicine Suginami Hospital), Kengo Tokunaga (Department of Preventive Medicine, Kyorin University School of Medicine), Shigemi Nakashima (Clinical Education Center for Medical Doctors, Shiga University of Medical Science), and Hidenori Matsui (Department of Bacteriology II, National Institute of Infectious Diseases).

**SR collaborators**

Kazunori Adachi (Department of Internal Medicine, Division of Gastroenterology, Aichi Medical University School of Medicine), Kazumi Inokuchi (Division of Gastroenterology and Hepatology, Department of Internal Medicine, Keio University School of Medicine), Takuma Okamura (Department of Gastroenterology, Shinshu University School of Medicine), Kazutake Oguma (Department of Internal Medicine, Asoka Hospital), Kosuke Sakitani (Bunkyo Dozaka Clinic), Tae Sasakabe (Department of Public Health, Aichi Medical University School of Medicine), Hiroshi Mihara (Department of General Medicine, Faculty of Medicine, Sapporo Medical University), Chikara Iino (Department of Gastroenterology and Hematology, Graduate School of Medicine, Hirosaki University), Yuichiro Ikebuchi (Division of Gastroenterology and Nephrology, Tottori University School of Medicine), Eri Iwata (Department of Gastroenterological Endoscopy, Tokyo Medical University Hospital), Sho Onodera (Department of Gastroenterology, Graduate School of Medicine, Yokohama City University), Yusaku Kajihara (Department of Gastroenterology, Murakami Hospital), Chihiro Kato (Division of Gastroenterology and Hepatology, Department of Internal Medicine, Keio University School of Medicine), Hiroki Sato (Asahikawa Medical University), Shu Sahara (Department of Gastroenterology, Hamamatsu Medical Center), Daisuke Suto (Department of Gastroenterology, International University of Health and Welfare Hospital), Ayaka Takasu (Division of Gastroenterology and Hepatology, Department of Medicine, Nihon University School of Medicine), Ryu Tanaka (Endoscopy Center, Shinjuku Tsurukame Clinic), Keisuke Hata (Department of Gastroenterology, Nihonbashi Muromachi Mitsui Tower Midtown Clinic), Akira Higashimori (Department of Gastroenterology, Osaka Metropolitan University Graduate School of Medicine), Tomohiro Higuchi (Department of Gastroenterology, Hamamatsu Medical Center), Takeshi Fujieda (Department of Internal Medicine, Kitaibaraki City Hospital), Horii Toshiki (Department of Gastroenterology, Kitasato University of Medicine), Murata Masaki (Department of Gastroenterology, National Hospital Organization Kyoto Medical Center), Kohei Morioka (Division of Gastroenterology and Hepatology, Department of Internal Medicine, Keio University School of Medicine), Takeshi Yasuda (Department of Gastroenterology, Akashi City Hospital), Hiroshi Kishikawa (Department of Gastroenterology, Tokyo Dental College, Ichikawa General Hospital, Chiba, Japan), Masaaki Kodama (Department of Advanced Medical Sciences, Oita University), Takako Sasai (Junpukai Health Maintenance Center, Okayama), Naoki Sumi (Department of Health Care Medicine, Kawasaki Medical School, Kurashiki), Daisuke Chinda (Division of Endoscopy, Hirosaki University Hospital, Aomori, Japan), Shinichi Horii (Taniko Hospital, Kiboukai Medical Corporation), Kazuo Yashima (Division of Gastroenterology and Nephrology, Tottori University School of Medicine), Hiroyuki Yamamoto (Department of Gastroenterology, Cancer Institute Hospital, Japanese Foundation for Cancer Research, Tokyo, Japan), Shuhei Yoshida (Department of General Internal Medicine, Hiroshima University Hospital), Yingsong Lin (Department of Public Health, Aichi Medical University), Yoshihiro Azuma (Department of Pediatrics, Yamaguchi University Graduate School of Medicine), Natsuki Ito (Department of Pediatrics, Juntendo University Faculty of Medicine), Takeshi Utsunomiya (Department of Pediatrics, Hyogo Medical University), Sayo Kawai (Department of Public Health, Aichi Medical University School of Medicine), Hiroaki Saito (department of Radiation health management, Fukushima medical university school of medicine), Mayu Sakai (Oita University Faculty of Medicine), Tomoki Sato (Department of Pediatrics, Hiroshima City Funairi Citizens Hospital), Yoshihiko Sugino (Department of Pediatrics, Japanese Red Cross Kumamoto Hospital), Masumi Nagata (Department of Pediatrics, Juntendo University Faculty of Medicine), Yoshitaka Nishikawa (Department of Health Informatics, Kyoto University Graduate School of Medicine and School of Public Health), Yuko Masuzawa (St. Luke’s International University, Graduate School of Nursing Science), and Masato Yoshiura (Department of Pediatrics, Faculty of Medicine, Saga University).
